# Cationic Cell-Penetrating Peptides Are Potent Furin Inhibitors

**DOI:** 10.1371/journal.pone.0130417

**Published:** 2015-06-25

**Authors:** Bruno Ramos-Molina, Adam N. Lick, Amir Nasrolahi Shirazi, Donghoon Oh, Rakesh Tiwari, Naglaa Salem El-Sayed, Keykavous Parang, Iris Lindberg

**Affiliations:** 1 Department of Anatomy and Neurobiology, School of Medicine, University of Maryland-Baltimore, Baltimore, Maryland, United States of America; 2 Chapman University, School of Pharmacy, Irvine, California, United States of America; University of Helsinki, FINLAND

## Abstract

Cationic cell-penetrating peptides have been widely used to enhance the intracellular delivery of various types of cargoes, such as drugs and proteins. These reagents are chemically similar to the multi-basic peptides that are known to be potent proprotein convertase inhibitors. Here, we report that both HIV-1 TAT_47-57_ peptide and the Chariot reagent are micromolar inhibitors of furin activity *in vitro*. In agreement, HIV-1 TAT_47-57_ reduced HT1080 cell migration, thought to be mediated by proprotein convertases, by 25%. In addition, cyclic polyarginine peptides containing hydrophobic moieties which have been previously used as transfection reagents also exhibited potent furin inhibition *in vitro* and also inhibited intracellular convertases. Our finding that cationic cell-penetrating peptides exert potent effects on cellular convertase activity should be taken into account when biological effects are assessed.

## Introduction

Cationic peptides present within envelope proteins are used by many viruses to gain entry into host cells. These peptides, which efficiently pass through the plasma membrane and either remain in the cytoplasm or reach the nucleus, are frequently used as protein transduction reagents (reviewed in [1,2]). The use of cell-penetrating peptides (CPPs) has even been proposed as a drug delivery tool for therapeutic molecules in various diseases, for example cancer [3]. One of the most studied CPPs over the past decade has been the human immunodeficiency virus type 1 (HIV-1) transcriptional activator, the TAT protein, a virally-encoded regulatory factor essential for viral replication [4]. Many different studies have now confirmed that the highly basic region located between residues 47–57 is necessary and sufficient for intracellular import and delivery of a variety of proteins and nucleic acids [3,5,6]. In addition to the TAT peptide, numerous natural and synthetic CPPs have been described in the literature (i.e. penetratrin [7], Pep-1/Chariot [8], and polyarginine-containing peptides [9,10,11]) and are now commercially available. Variants on this theme include certain cyclic polyarginine peptides with high cell permeability and stability which have been recently used for the delivery of a wide range of cargoes, including anticancer and antiviral drugs; and phosphopeptides [12,13,14].

The proprotein convertase (PC) furin is a ubiquitous calcium-dependent endoprotease that is involved in the cleavage of a variety of precursor proteins at strings of basic amino acids within the constitutive secretory pathway. Polyarginines are known to constitute potent inhibitors of furin and other members of the family of the proprotein convertases. For example, hexa-D-arginine amide (D6R) and nona-D-arginine amide (D9R) exhibit inhibition constants against furin and other convertases in the nanomolar range [15,16]. In agrement, polyarginine-based peptides have been shown to block furin-mediated activation of various bacterial toxins, both *in vivo* and *in vitro* [17,18,19,20,21]. Molecular modeling studies support the idea that polyarginine binding is likely mediated by the acidic substrate binding cleft within the furin catalytic domain [15].

In order to assess the possibility that CPPs used for the intracellular delivery of proteins and drugs might exert side effects on cellular proprotein convertases, in the study reported below we have investigated their inhibitory effects on convertase activity, both *in vitro* and within cells.

## Materials and Methods

### Materials

Soluble human furin was purified from the conditioned medium of stably-transfected, methotrexate-amplified CHO DG44 cells, as previously described [15]. Nona-D-arginine amide (D9R) was synthesized by Pepceuticals (New Orleans, LA) and purified by reverse-phase HPLC to greater than 99% purity. The HIV-1 TAT_47-57_ peptide was purchased from Creative Peptides (Shirley, NY). The Chariot reagent was purchased from Active Motif (Carlsbad, CA). The Chariot and HIV Tat peptides were not terminally blocked. All cyclic polyarginine peptides used in this work ([W_5_R_4_C], [WR]_5_, C_12_-[R_5_], and W_4_-[R_5_]) were synthesized using a Fmoc/*t*Bu solid-phase peptide synthesis strategy according to a previously described procedure [13,22]. The first two peptides ([W_5_R_4_C]; [WRWRWRWRWC]) and ([WR]_5_; [WRWRWRWRWR]) are cyclic and thus have no N- and C- terminal modifications. The third peptide (C_12_-[R_5_]; dodecanoyl-[KRRRRR]) is also cyclic and does not contain N or C-terminal modifications. The fourth peptide (W_4_-[R_5_]; N-acetyl-WWWW-[KRRRRR]) is N-terminally acetylated.

### Enzyme assays and determination of Ki values

The furin assay was performed in 96-well polypropylene microtiter plates in a final volume of 50 μl, containing 100 mM HEPES, pH 7.0, 5 mM CaCl_2_, 0.1% Brij 35, 0.1% NaN_3_, and 0.1 mg/ml BSA. The substrate p-Glu-Arg-Thr-Lys-Arg-4-methylcoumaryl-7-amide (pERTKR-mca; Peptide Institute, Lexington, KY) was used at a final concentration of 100 μM. Furin was used at a final concentration of 20 nM. Reaction mixtures were incubated at 37°C and fluorescence measurements (380 nm excitation, 460 nm emission) were taken under kinetic conditions every minute for 60 min in a SpectraMax M2 microplate reader. For Ki assays, serial dilutions of compounds were performed to give final concentrations between 10 nM and 10 μM in 50 μl. After a 30-min preincubation at room temperature, 100 μM of pERTKR-methylaminocoumarin (mca) was added, and residual enzyme activities were monitored by measuring mca fluorescence intensity. Data were analyzed using Prism 5 as described previously [23]. Due to cost considerations, Ki determinations were not performed for the HIV TAT peptide or for the Chariot reagent.

### Cell migration assays

HT1080 fibrosarcoma cells (ATCC# CCL-121) were cultured to 80% confluence in growth medium (MEM (Earle’s salts + L-glutamine) 10% FBS, 1:100 non-essential amino acids (NEAA), 1 mM sodium pyruvate, 500 U/ml penicillin-streptomycin, and 1% gentamycin; Life Technologies). Cells were plated in an Oris Cell Migration Assay (Platypus Technologies) 96-well plate at 10^5^ cells per well, following the manufacturer’s protocol. The next day, the growth medium was removed, the wells rinsed with PBS, and the cells were incubated in assay medium MEM (Earle’s salts + L-glutamine) containing 10% heat-inactivated FBS, 1:100 NEAA, 1 mM sodium pyruvate, 500U/ml penicillin-streptomycin, and 1% gentamycin in the presence or absence of inhibitors for 24 h at 37°C and 5% CO_2_. After incubation cells were rinsed with PBS (calcium and 20 mM HEPES, pH 7.4, as per the manufacturer’s protocol), and incubated with the Live / Dead Cell Stain Kit containing 2 μM calcein AM and 4 μM ethidium homodimer (EthD-1) for 30 min at 37°C and 5% CO_2_. Fluorescence was then measured at 485/528 nm excitation/emission for calcein AM, and 530/645 nm for EthD-1. The experiments were independently repeated three times.

### Cytotoxicity assay

In order to assess the potential cytotoxic effects of each compound, cytotoxicity assays were performed in CHO DG44 cells (obtained from Lawrence Chasin, Columbia University and grown in Ham’s F12 medium with 10% bovine serum) using the mitochondrial dye WST-1 (Roche). Cells were seeded into 96-well plates to achieve 50% confluence the next day, and then incubated with each compound or with vehicle for 24 h. After incubation with inhibitors, cells were further incubated for 4 h with 10 μl of WST-1 reagent per well, and the absorbance was measured at 450 and 600 nm. The experiments were repeated independently 2–3 times using triplicate wells.

### SEAP activity assays

CHO-GRAPfurin cells expressing the hybrid reporter protein GRAPfurin, consisting of the secreted alkaline phosphatase (SEAP) protein fused to a Golgi retention signal and a specific furin recognition/cleavage site [24,25], were plated in 96-well plates and incubated with OptiMem containing 100 μM of either drug or vehicle for 16–20 h. The medium was collected, centrifuged, and heated for 30 min at 65°C to inactivate non-relevant phosphatases. To test SEAP activity, 2.5 μl of heated medium was mixed with 100 μl of assay buffer (100 mM Tris-HCl, pH 10, 100 mM NaCl, 5 mM MgCl_2_) and 100 μl of 36 μM 4-methylumbelliferyl phosphate (MUP), a phosphatase substrate, made in 50 mM Tris-HCl, pH 10. Fluorescence was measured every 20 seconds after excitation at 365 nm and recording emission at 460 nm at 37°C for 1 h. Since SEAP released from the tethered furin reporter is secreted, SEAP levels in the medium are proportional to the activity of Golgi furin [24,25]. The experiments were independently repeated three times using triplicate wells per condition.

## Results

### The HIV-1 TAT_47-57_ and Chariot peptides inhibit furin activity *in vitro*

**Fig 1:**
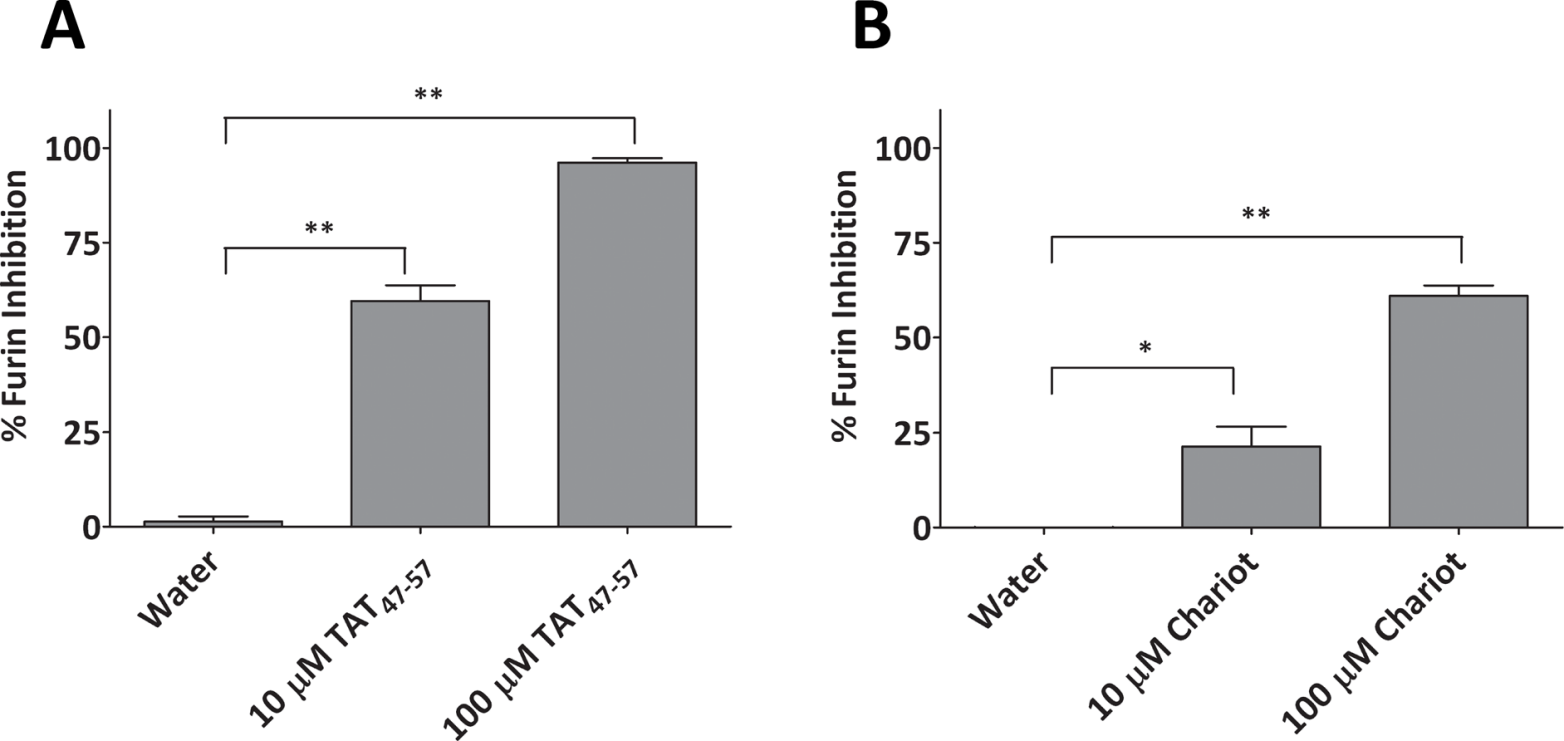
Inhibition of furin by the cationic peptides HIV-1 TAT_47-57_ and Chariot. Soluble human furin, pre-incubated for 20 min at room temperature in the presence of (a) HIV-1 TAT (47–57) or (b) Chariot peptide, was tested at the specified concentrations. Furin activity was assessed by measuring the release of the fluorescent mca product from the fluorogenic substrate, pERTKR-mca. Results represent the mean ± S.D., N = 3. *P<0.01; **P<0.05.

To determine the effect of the polybasic carrier peptide HIV-1 TAT_47-57_ and the Chariot transfection reagent (Table 1) on furin activity, we performed *in vitro* enzyme assays. The peptides were preincubated with soluble human furin in assay buffer and then further incubated with the fluorogenic substrate pERTKR-mca, as described in “Materials and Methods”. Fig 1A shows that the HIV-1 TAT_47-57_ peptide produced substantial furin inhibition at micromolar concentrations (~60% at 10 μM). The inhibition of furin activity was nearly complete at the higher concentration of 100 μM (Fig 1A). The Chariot reagent also inhibited furin at micromolar concentrations (~20% at 10 μM; ~60% at 100 μM), although much less potently than the HIV-1 TAT_47-57_ peptide (Fig 1B). This difference may be attributable to the greater number of arginine residues present in the HIV-1 TAT_47-57_ peptide sequence (Table 1). It should be noted that the amounts of Chariot reagent used in these assays are within the range of the manufacturer’s suggestions for use as a protein transfection adjuvant (10 μM to 100 μM).

**Table 1:** Cationic cell-penetrating peptides tested as furin inhibitors.

Name	Origin	Sequence
TAT_47-57_	HIV-1 protein	YGRKKRRQRRR
Chariot	Synthetic	KETWWETWWTEWSQPKKKRKV

### HIV-1 TAT peptide inhibits cancer cell migration

**Fig 2:**
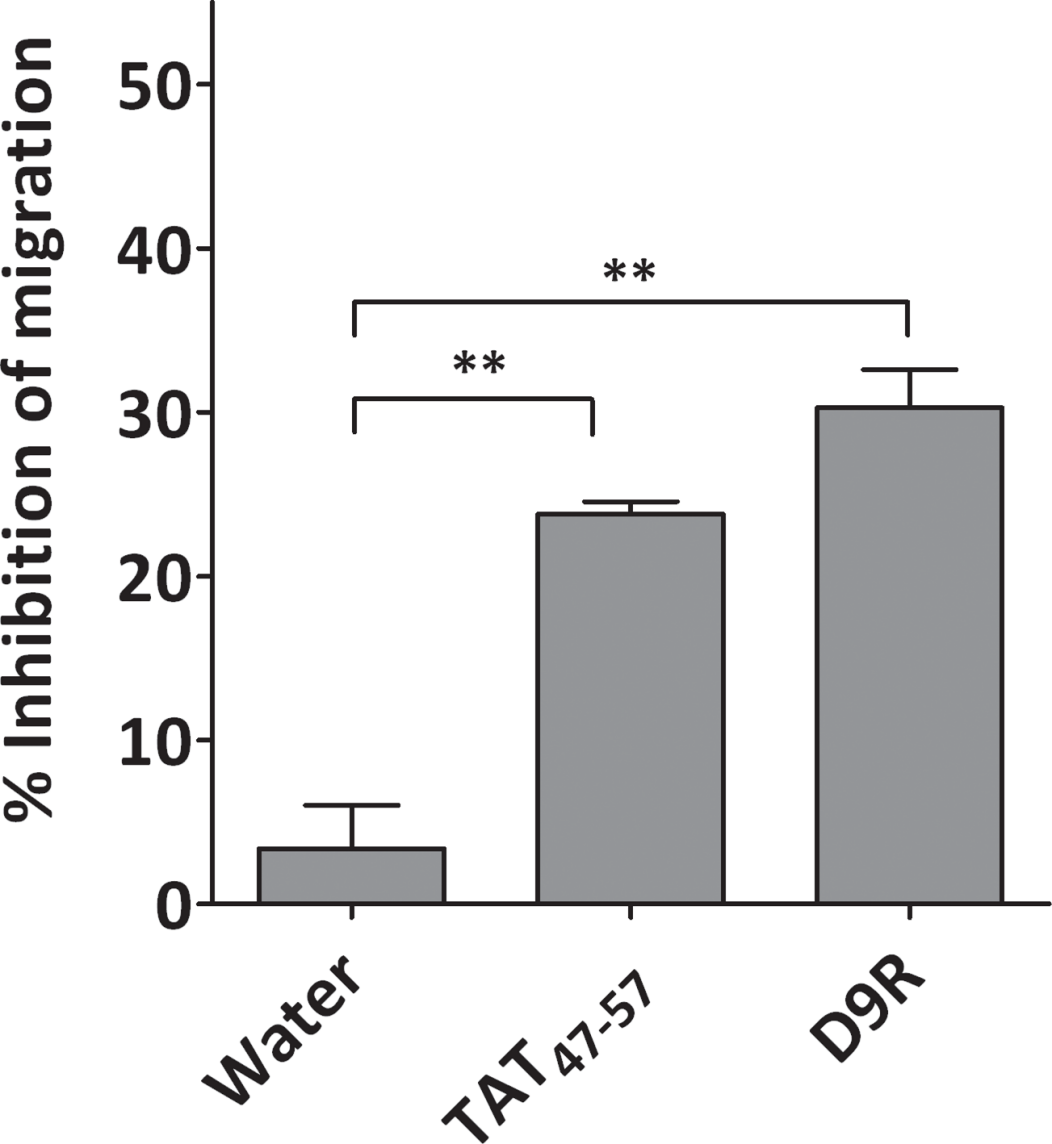
Effect of HIV-1 TAT_47-57_ peptide on cell migration of fibrosarcoma HT1080 cells. Cells were incubated with either 10 μM of peptide or vehicle (water) for 24 h at 37°C and cell migration was measured as described in “Materials and Methods”. Results represent the mean ± S.D., N = 3. **P<0.05.

Because of its inhibitory potency *in vitro* against furin, as well as its known cell permeability, we then analyzed the inhibitory capacity of the HIV-1 TAT_47-57_ peptide against cancer cell migration, a process dependent on the activity of cellular convertases. We incubated HT1080 fibrosarcoma cells together with a non-toxic quantity of the HIV-1 TAT_47-57_ peptide (10 μM). Fig 2 shows that incubation of cells with HIV-1 TAT_47-57_ resulted in significant inhibition of cell migration, similar to that obtained with the multi-Leu convertase inhibitor peptide [26,27].

### Cyclic polyarginine peptides are potent furin inhibitors *in vitro*

**Fig 3:**
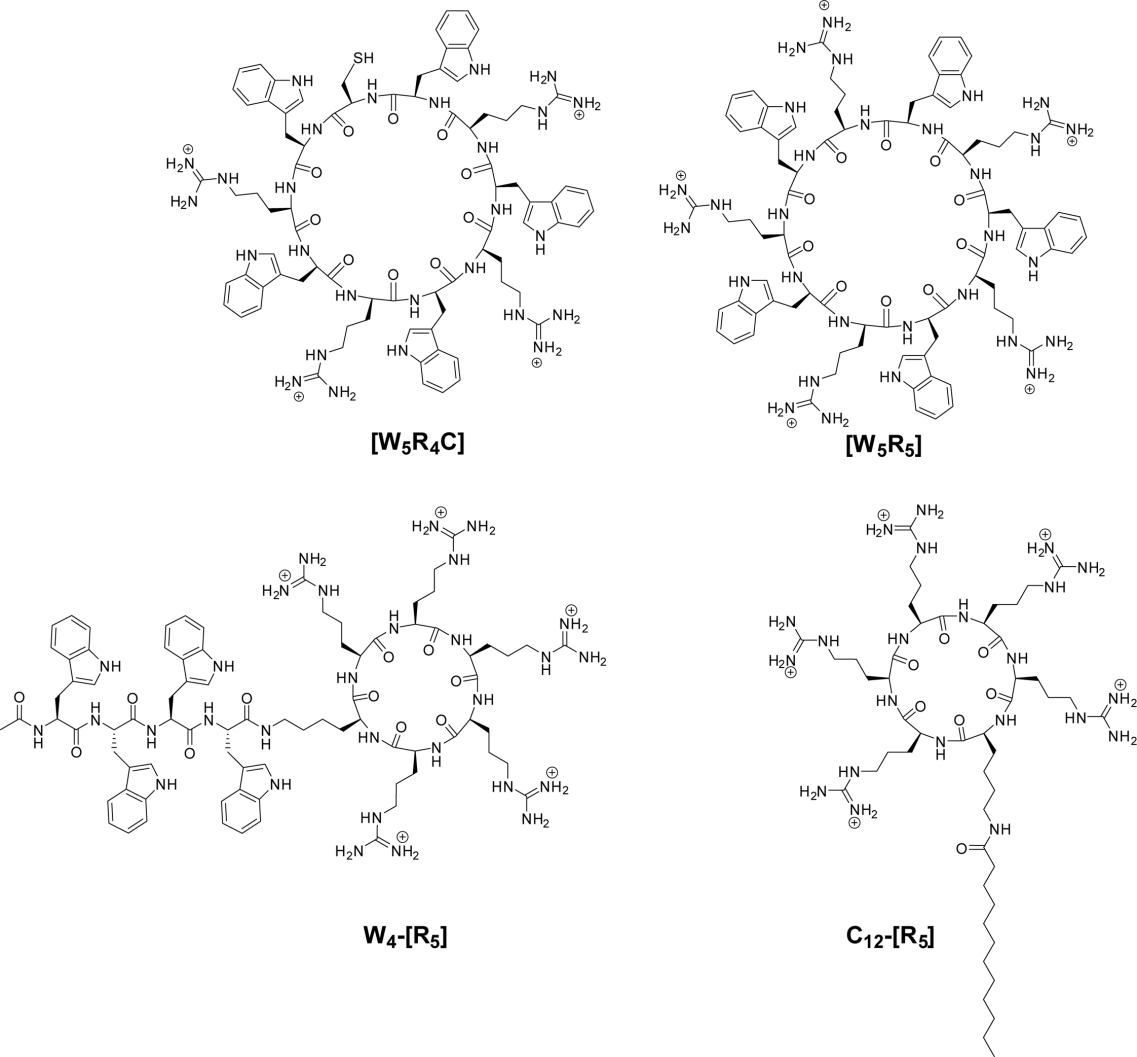
Chemical structures of cyclic polyarginine peptides tested as furin inhibitors.

Stable and cell-permeable cyclic polyarginine peptides, such as the C_12_-[R_5_] compound, have been reported to exhibit little cytotoxicity [13,14]. Given the known inhibition of furin activity by polyarginines [28] we examined the inhibitory capacity of these cyclic compounds on furin activity *in vitro*. The structures of the cyclic polyarginine peptides tested in this work are shown in Fig 3. These compounds exhibited high inhibitory potency *in vitro*, with Ki values between 1 μM and 0.1 μM (Table 2).

**Table 2:** K_i_ values of the synthetic cyclic peptides tested as furin inhibitors.

Name	Peptide sequence	Ki (μM)
[W_5_R_4_C]	WRWRWRWRWC	0.34 ± 0.02
[WR]_5_	WRWRWRWRWR	0.98 ± 0.14
C_12_-[R_5_]	Dodecanoyl-[KRRRRR]	1.02 ± 0.40
W_4_-[R_5_]	N-acetyl-WWWW-[KRRRRR]	0.10 ± 0.14
Hexa-D-arginine	rrrrrr-amide	0.106 ± 0.010
Nona-D-arginine	rrrrrrrrr-amide	0.0013 ±0.002

The data for hexa-D-arginine (D6R) and nona-D-arginine (D9R) are taken from [28] and [15] respectively.

### Cyclic polyarginine peptides inhibit intracellular convertases

**Fig 4:**
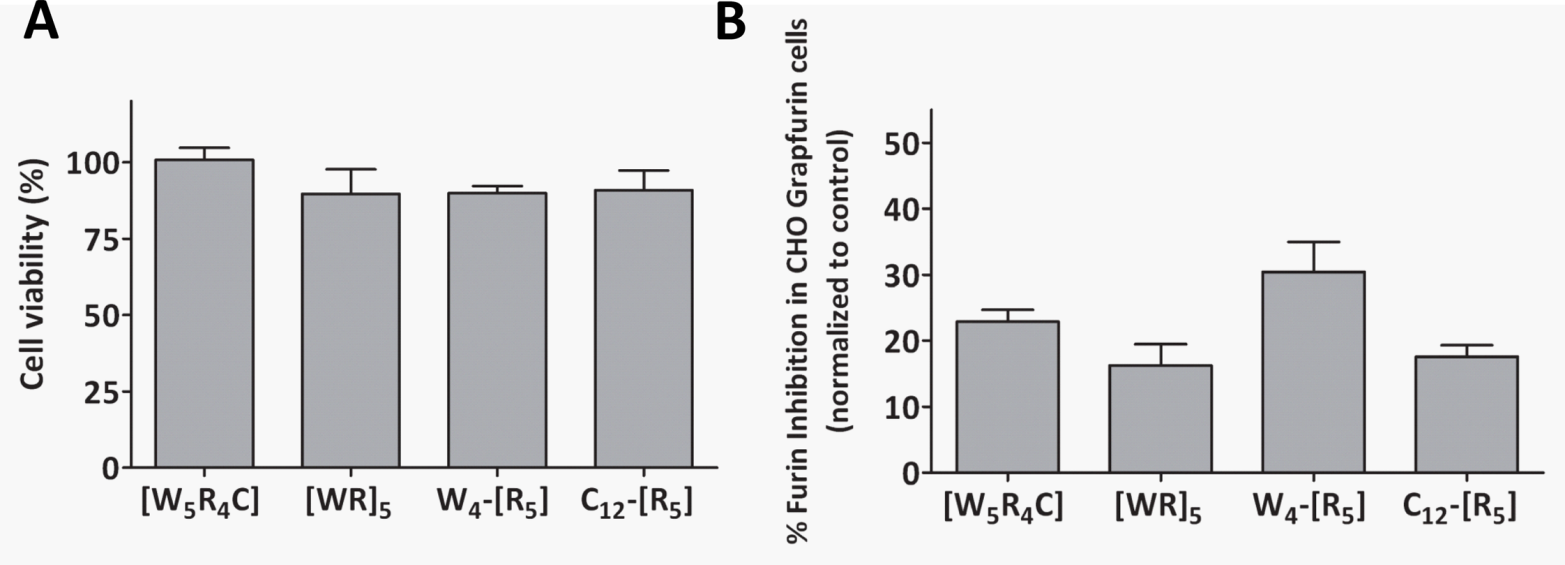
Cyclic polyarginine peptides inhibit cellular convertase activity. (a) CHO cells were incubated with each compound at 1 μM for 24 h at 37°C, and cell viability was monitored by incubation for 4 h with WST-1. (b) CHO-GRAPfurin cells, expressing secreted alkaline phosphatase tethered to Golgi membranes by a transmembrane domain interrupted by a furin cleavage site, was incubated with 1 μM of each cyclic peptide for 20–24 h at 37 °C. Secreted alkaline phosphatase activity was measured in the medium. Results represent the mean ± S.D., N = 3.

In agreement with a previous study [13], we found no cytoxicity after a 24-h incubation of CHO cells with cyclic polyarginine compounds at 1 μM (Fig 4A). Interestingly, all compounds exhibited significant inhibition of intracellular convertase activity in the TGN at this concentration, as demonstrated using an assay based on the release of SEAP from a Grap-furin Golgi reporter tethered to membranes via a furin consensus cleavage site [24] (Fig 4B). While this assay is not specific for furin—as opposed to other constitutively-expressed convertases such as PACE4—these SEAP assay results correlate well with inhibition results obtained *in vitro*, as the most effective compound in cells (W_4_-[R]_5_) was also the most potent furin inhibitor *in vitro* (Table 2).

## Discussion

Cationic cell-penetrating peptides (CPPs) have been broadly used for the delivery of various types of molecular cargoes such as small molecule drugs, siRNAs, and phosphopeptides (reviewed in [1,2,29]). Most of these compounds contain a polybasic domain responsible for transport into the intracellular space. The initial, and still the best characterized CPP, is the trans-acting activator of transcription (TAT) peptide derived from the human immunodeficiency virus [30,31]. Exhaustive analyses have demonstrated that the sequence responsible for its cellular uptake consists of the arginine-rich region YGRKKRRQRRR located between residues 47 and 57 [3,29]. The relevance of the arginine residues to uptake was clearly demonstrated by the assay of truncated analogs of HIV-1 TAT_47-57_ [10]. The practical applications of the use of this peptide *in vivo* have been previously established [32]. In this latter study, Schwarze and colleagues injected a fusion protein composed of HIV-1 TAT_47-57_ and β-galactosidase intraperitoneally into mice, and subsequently detected significant local β-galactosidase activities in most of the tissues analyzed. Aside from HIV-1 TAT_47-57_, a variety of other polyarginine-containing peptides have been proposed for the intracellular delivery of nucleic acids, proteins, and drugs [33,34]. Indeed, several groups have proposed the use of cationic transfection peptides as a means of delivering therapeutic species in the treatment of human diseases such as cancer [3,35].

Simple arginine-rich peptides themselves have been also proposed for use as transfection reagents since they enter cells efficiently [9,10,36,37,38]. However, polyarginine-containing peptides are known to potently inhibit several members of the proprotein convertase (PC) family, such as furin, PC5/6, PACE4 and PC7 [16,28,39,40,41]. The results shown here strongly support the idea that the HIV-1 TAT_47-57_ peptide and Chariot transfection reagent do possess the off-target effect of inhibiting furin (and likely other proprotein convertases). Interestingly, we show here that the TAT_47-57_ CPP also inhibits cancer cell migration. These results can be potentially be linked to effects on cellular convertase activity, since numerous studies have described furin-mediated activating cleavage of certain metalloproteinases, i.e. stromelysin-3 and proMT1-MMP, whose activation then results in extracellular matrix degradation [25,42,43,44]. The convertase-inhibiting property of the HIV-1 TAT_47-57_ peptide might in fact assist the therapeutic efficacy of any delivered anticancer cargo via the inhibition of the elevated intracellular convertase activity known to be associated with tumor development and metastasis (reviewed in [45]).

In addition to the linear CPPs, a number of synthetic cyclic polyarginines with efficient cell permeability have also been recently proposed as CPPs to assist the intracellular delivery of proteins, drugs and nucleic acids [12,14,46,47,48,49,50]. Our results show that these cyclic polyarginines also represent potent inhibitors of furin activity *in vitro*. Similar to previous studies [13,14], the treatment of cells with cyclic polyarginines for 24 h was not cytotoxic. In agreement with their efficient uptake and likely low rates of intracellular degradation, these cyclic compounds all inhibited intracellular convertases, as assessed by blockade of the release of a furin cleavage reporter molecule. Cyclic polyarginines may thus be of use in applications where intracellular furin inhibition is advantageous, such as the prevention of tumor cell proliferation and migration mentioned above.

Off-target effects of cationic CPPs (i.e. on biological activities other than transport) have been previously cataloged in a recent review [2] and include a variety of biological effects, such as oxidative stress effects, responsiveness to heparan sulfate, lipid remodeling, and actin rearrangement. Interestingly, the only prior study that has addressed the interaction of CPPs with proprotein convertases concluded that furin may act to inactivate the TAT_7-57_ peptide, although furin-mediated inactivation was not directly demonstrated in this work [51]. Our data support a contradictory conclusion: that TAT4_7-57_ acts to inhibit intracellular furin and/or other convertases.

In conclusion, the data presented here demonstrate that a variety of cell-penetrating peptides (HIV-1 TAT_47-57_, Chariot, and cyclic polyarginine peptides) which are widely used as protein transduction agents can significantly inhibit cellular convertase activity. While not necessarily deleterious (for example in anti-cancer applications; [45]), this off-target effect must be taken into account in *in vivo* therapeutic applications of polyarginine-containing CPP compounds.
